# Identification of Broad-Spectrum Antiviral Compounds by Targeting Viral Entry

**DOI:** 10.3390/v11020176

**Published:** 2019-02-20

**Authors:** Michela Mazzon, Ana Maria Ortega-Prieto, Douglas Imrie, Christin Luft, Lena Hess, Stephanie Czieso, Joe Grove, Jessica Katy Skelton, Laura Farleigh, Joachim J. Bugert, Edward Wright, Nigel Temperton, Richard Angell, Sally Oxenford, Michael Jacobs, Robin Ketteler, Marcus Dorner, Mark Marsh

**Affiliations:** 1 MRC Laboratory for Molecular Cell Biology, University College London, London WC1E 6BT, UK; douglas_imrie@hotmail.co.uk (D.I.); c.luft@ucl.ac.uk (C.L.); lena.hess@meduniwien.ac.at (L.H.); s.czieso@imperial.ac.uk (S.C.); r.ketteler@ucl.ac.uk (R.K.); 2 Section of Virology, Department of Medicine, School of Medicine, Imperial College London, London W2 1PG, UK; a.ortega-prieto@imperial.ac.uk (A.M.O.-P.); j.skelton15@imperial.ac.uk (J.K.S.); m.dorner@imperial.ac.uk (M.D.); 3 Institute of Immunity and Transplantation, Royal Free Hospital, University College London, London NW3 2QG, UK; j.grove@ucl.ac.uk; 4 Medical Microbiology, Cardiff University School of Medicine, Cardiff CF14 4XN, UK; laurafarleigh@hotmail.com (L.F.); Joachim1Bugert@bundeswehr.org (J.J.B.); 5 Bundeswehr Institute of Microbiology, 80937 Munich, Germany; 6 University of Sussex, Brighton BN1 9RH, UK; ew323@sussex.ac.uk; 7 Medway School of Pharmacy, University of Kent, Chatham ME4 4TB, UK; N.Temperton@kent.ac.uk; 8 School of Pharmacy, University College London, London WC1N 1AX, UK; r.angell@ucl.ac.uk (R.A.); s.oxenford@ucl.ac.uk (S.O.); 9 Faculty of Medical Sciences, UCL Medical School, London NW3 2QG, UK; michael.jacobs@ucl.ac.uk

**Keywords:** broad-spectrum antivirals, host-targeted antivirals, alphaviruses, flaviviruses, endocytosis, virus entry, Zika, dengue, Semliki Forest virus, phenotypic screening

## Abstract

Viruses are a major threat to human health and economic well-being. In recent years Ebola, Zika, influenza, and chikungunya virus epidemics have raised awareness that infections can spread rapidly before vaccines or specific antagonists can be made available. Broad-spectrum antivirals are drugs with the potential to inhibit infection by viruses from different groups or families, which may be deployed during outbreaks when specific diagnostics, vaccines or directly acting antivirals are not available. While pathogen-directed approaches are generally effective against a few closely related viruses, targeting cellular pathways used by multiple viral agents can have broad-spectrum efficacy. Virus entry, particularly clathrin-mediated endocytosis, constitutes an attractive target as it is used by many viruses. Using a phenotypic screening strategy where the inhibitory activity of small molecules was sequentially tested against different viruses, we identified 12 compounds with broad-spectrum activity, and found a subset blocking viral internalisation and/or fusion. Importantly, we show that compounds identified with this approach can reduce viral replication in a mouse model of Zika infection. This work provides proof of concept that it is possible to identify broad-spectrum inhibitors by iterative phenotypic screenings, and that inhibition of host-pathways critical for viral life cycles can be an effective antiviral strategy.

## 1. Introduction

Emerging and re-emerging viruses present a major threat to human and animal health. In recent years epidemics of Ebola [1], Zika [2], yellow fever [3], influenza [4], and chikungunya [5] viruses amongst others have raised awareness that viruses can spread rapidly and extensively before vaccines can be developed or made available to affected populations.

The development of virus-specific vaccines or directly-acting antivirals (DAA) can be a lengthy process, fraught with difficulties and challenges. In the face of a new viral outbreak, it is rare for a new vaccine or DAA to be made available for clinical use in time to make a difference.

Broad-spectrum antivirals (BSA) are drugs with the potential to inhibit infection by multiple viruses from different groups or families [6,7]. BSA are attractive because they can be deployed rapidly during epidemics of emerging or re-emerging viruses without the need for accurate diagnostics, or when specific vaccines or therapies are not available. Also, by targeting cellular rather than viral components, the risk of selecting for viral resistance is much lower [8].

Given the broad diversity of viruses, even within families, pathogen-directed approaches that target viral proteins are unlikely to have broad-spectrum efficacy. Although many studies of virus–host interaction have revealed a great complexity and divergence in the way these pathogens manipulate infected cells, there are a number of conserved cellular pathways that are exploited by many different viruses [7,8]. Pharmacological inhibition of any one of these pathways would then be expected to reduce infection by viruses that use that specific host machinery.

In this context, virus entry constitutes an attractive target. While different viruses may use different cellular cues to facilitate access to the cytoplasm of a target cell, only a limited number of entry routes exists, suggesting that by inhibiting one pathway it might be possible to inhibit infection by all viruses that depend on that mechanism [8,9]. Clathrin-mediated endocytosis (CME) in particular is widely used by many small enveloped RNA viruses, including alpha-, flavi-, and influenza viruses [10,11]. After adhesion to the cell surface and binding to specific cellular receptors, all these viruses are internalised in clathrin-coated vesicles that fuse with endosomes. Within endosomes, a drop in pH can induce conformational changes in the viral envelope proteins, which initiate viral fusion with endosomal membranes and release of the viral RNA into the host cell cytoplasm [10,11]. Low pH also plays a role in the fusion of other enveloped viruses, although the exact mechanism of endocytosis may vary [12,13].

While the notion of BSA targeting cellular pathways has been previously proposed, few studies have led to the identification and full in vitro and in vivo characterisation of such compounds. In this study we developed an iterative phenotypic screening strategy to identify small molecules with broad-spectrum potential, and verify their activity on multiple viruses. Finally we tested the activity of the most potent and broad-spectrum of these compounds in a mouse model of Zika virus (ZIKV) infection and observed a reduction of viral replication in vivo.

## 2. Materials and Methods

### 2.1. Viruses, Virus-Like Particles (VLPs), and Infection

Semliki Forest virus (SFV)_Zs-Green (kind gift of Giuseppe Balistreri, University of Helsinki, described in [14]), Sindbis virus (SINV, AR339 strain, a kind gift of Penny Powell, University of East Anglia), and Ross River virus (RRV, a kind gift of Suresh Mahalingham, Griffith University) were expanded and titrated by plaque assay on BHK cells [15]. All serotypes of dengue virus (DENV, a kind gift of Andrew Davidson, University of Bristol) were expanded in C6/36 cells and titrated by immunofocus assay on Vero cells. Zika virus (ZIKV) and yellow fever virus (YFV) 17D were expanded and titrated by immunofocus assay on Vero cells. Hepatitis C virus (HCV J6/JFH) was expanded and quantified in Huh7.5 cells. The Nef-negative human immunodeficiency virus (HIV)-1 molecular clone NL4.3-R3A was provided by Jim Hoxie (UPenn, Philadelphia, USA). Virus was produced in HEK-293T cells as described [16] and titrated in HeLa TZMbl. Vaccinia virus (VACV) Western Reserve-GFP, (a kind gift of Jason Mercer, MRC LMCB, UCL) was expanded and titrated in BSC-1 cells. Herpes simplex virus (HSV-1, kind gift of Richard Milne, UCL) was expanded and quantified in Vero cells.

Vesicular stomatitis virus (VSV), Rabies virus (RABV), influenza H5N1, and Ebola virus (EBOV) virus-like particles (VLPs) were generated by co-transfection of pCMV-d8.91 [17], incorporating the HIV-1 gag-pol gene enabling the formation of VLP cores (1.5 μg in one 10 cm^2^ dish) with pHR-SIN CSGW PGK Puro encoding eGFP (3.2 μg/dish), and either VSV-G (pMD2.G plasmid from Addgene, https://www.addgene.org/12259/), RABV-G (pI.18 plasmid, encoding the RABV isolate CVS-11 (ATCC VR-959) [18]), influenza HA (pI.18 plasmids encoding H5A/Vietnam/1194/2004 (Gene Bank accession number EF541402.1/ABP51976)) and NA A/PuertoRico/8/1934 (Protein Data Bank ID ode 1RU7) [19], or EBOV Sudan glycoprotein (pCAGGS [20] SUDV, GP isolate Boniface/SUD/1976, FJ968794) expressing plasmids (1.5 μg/dish) in HEK 293T cells (1.3 million cells plated the day before) using Fugene 6 (Promega, UK) according to manufacturer’s instructions. VSV_Blam-pseudotyped VLPs were generated by co-transfecting pCMV-d p8.91, the plasmid expressing VSV-G and a plasmid expressing Blam-tagged HIV-1 Vpr (HIV-1 YU2 Vpr β-lactamase Expression Vector (pMM310)). EBOV_Blam-pseudotyped VLPs were generated by co-transfecting the plasmid expressing EBOV Sudan glycoprotein and a plasmid expressing Blam-tagged VP40, as described previously [21].

Infections with SFV, SINV, and RRV were carried out in Dulbecco’s modified Eagle’s medium DMEM_ (Gibco, Thermo Fisher Scientific, UK) supplemented with 1% p/s (100× Gibco), 0.2% bovine serum albumin (from 7.5% solution, Gibco), and 10 mM Hepes. Infections with all other viruses or VLPs were performed in media compatible with the cells of choice supplemented with 2% fetal bovine serum (FBS), and 1% p/s. 

### 2.2. Cells

HeLa Kyoto (a kind gift of Marino Zerial, Max Planck Institute of Molecular Cell Biology and Genetics, Dresden), HeLa TZM-bl (a kind gift of Juan Martin-Serrano, King’s College London), Huh7 (a kind gift of Andrew Davidson, University of Bristol), and Huh7.5 (Apath LLC) cells were cultured in DMEM supplemented with 10% FBS and 1% penicillin/streptomycin (p/s); Vero cells (a kind gift of Andrew Davidson, University of Bristol) were cultured in M199 media supplemented with 5% FBS and 1% p/s; A549 cells (a kind gift of Paul Kellam, Sanger Institute, Cambridge) were cultured in F12 media (Gibco), supplemented with 10% FBS and 1% p/s.

### 2.3. Compounds

The screening was performed using the Sigma LOPAC (Library of Pharmacologically Active Compounds) and the Prestwick chemical libraries (each including 1260 compounds). For follow-up studies, compounds were all re-purchased from Sigma, with the exception of Raloxifene (Tocris Biosciences, UK). Purity and identity of the repurchased compounds was confirmed by liquid chromatography mass spectrometry (LC-MS). Sofosbuvir was purchased from Selleckchem (Germany).

### 2.4. Phenotypic Screening

HeLa Kyoto cells (8000 cells/well) were plated in 100 μL volume in each well of Perkin Elmer ViewPlates 96-black. The day after, the culture media was removed and replaced with 10 μM compounds in 100 μL of supplemented DMEM. 45 min later, SFV_Zs-Green was added to each well at an MOI of 1, in 5 μL volumes, in the presence of compounds. In each plate, 4 wells of DMSO uninfected or infected cells were included as controls. As a positive control 8 wells of cells treated with 10 μM of Monensin before and during infection were also included.

Seven hours after infection, cells were washed in phosphate buffered saline (PBS), fixed with 4% paraformaldehyde (PFA) for 30 min, and stained with 5 μg/mL Hoechst before imaging on a Perkin Elmer (PE) Opera LX system. For each well, 9 images were acquired using a 20× objective. Total cell numbers and Zs-Green infected cells were counted using the PE Columbus software (Columbus 2.4.1.110801, PE, UK). Further analysis was performed using Microsoft Excel. For hit compound identification, results were normalised across all screened plates and z-scores calculated using cellHTS2 for R.

The secondary screening against DENV serotype 2 was performed as above, but in Huh7 cells and 2% FBS DMEM, letting the infection progress for 24 h in the presence of compounds, consistent with the longer DENV replication time compared with SFV. After fixation as above, cells were stained for DENV E protein, as described below.

### 2.5. Immunofluorescence Staining

After fixation, PFA was quenched with 50 mM ammonium chloride for 20 min at room temperature (RT). Following a wash in PBS, cells were permeabilised for 10 min at RT with 0.1% Triton X100 dissolved in washing buffer (0.5% bovine serum albumin/PBS). Permeabilisation buffer was removed and replaced with the appropriate concentration of primary antibody diluted in washing buffer for 90 min at RT. Following 3 washes in washing buffer, appropriate concentrations of secondary antibody and Hoechst 33342 (5 μg/mL) diluted in washing buffer were added to the cells for 60 min, before further washing. Washing buffer was replaced with 100 μL PBS for imaging.

The following antibodies were used: DENV-1, DENV-2, and DENV-3: mouse anti DENV 1–4 Envelope protein antibody (Clone D1-11(3), MA1-27093, Thermo Fisher Scientific, UK); ZIKV: 4G2 mouse anti-flavivirus antibody (MAB10216, Millipore, UK); SINV: mouse anti-SINV antibody, a kind gift from Penny Powell, University of East Anglia; RRV: mouse anti-alphavirus antibody (3581) (sc-58088, Santa Cruz, Dallas, Texas, US); YF17D: anti-yellow fever antibody (3576) (sc-58083, Santa Cruz, Dallas, Texas, US); HCV: anti-NS5 S38 hybridoma supernatant used at 1/100 (kind gift from Jane McKeating, University of Birmingham); HSV-1: anti HSV-1 ICP4 antibody (ab6514, Abcam, UK); goat anti-mouse Alexa 488 (A-11001, Thermo Fisher Scientific, UK); goat anti-rabbit 488 (A-11034, Thermo Fisher Scientific, UK).

### 2.6. MTT Assay

Cells (8000 cells/well) were plated in 100 μL volume in each well of a clear 96 plate in complete media. The following day, the cells were treated with the desired concentrations of drugs in 100 μL/well of supplemented DMEM (HeLa Kyoto) or 2% DMEM (Huh7) for a time equivalent to the duration of the drug treatment during the infection experiments. MTT (Thiazolyl Blue Tetrazolium Bromide, Sigma, UK) was diluted to 5 mg/mL in PBS and, after 7 h or 24 h, depending on the duration of the infectivity assay, 20 μL added to each well for 2 h at 37 °C. After washing, media were replaced with 40 μL/well of a 1:1 mix of DMSO:Isopropanol for 20 min at 37 °C; 35 μL were transferred to a clean plate and read at 570 nm.

### 2.7. IC_50_ and TC_50_ Calculation

Percentages of inhibition were calculated in Microsoft Excel, relative to DMSO uninfected (0%) and infected (100%) controls, using the following formula: 100 × (1 − ((% Infection Sample − % Infection Uninfected control)/(% Infection Infected control − % Infection Uninfected control))). IC_50_ and TC_50_ were calculated in GraphPad Prism, using a non-linear regression curve (log inhibitor vs. normalised response–variable slope).

### 2.8. β-Lactamase Assay

HeLa Kyoto cells (50,000 cells/well) were plated in 24-well plates. The following day, media was replaced with drugs diluted to 10 μM (or 20 μM for N-p Tosyl-L-PCK, Supercynnamaldehyde, and Raloxifene) for 1 h at 37 °C. 5 μL of VSV-G or EBOV_Sudan pseudotyped Blam-VLPs were added to each well in the presence of compounds and incubated for 2 h at 37 °C. Next, β-lactamase substrate prepared according to manufacturer’s instructions (LiveBLAzerTM FRET—B/G Loading Kit, Invitrogen, Thermo Fisher Scientific, UK) was added for 1 h, at room temperature (RT) in the dark. Next, substrate was removed and cells incubated in CO_2_-independent media supplemented with 2.5 mM Probenecid overnight (O/N), RT in the dark. The following day cells were washed, scraped in PBS, fixed with 1% PFA and analysed on an LSR-II (BD) flow cytometer, using the fluorescein isothiocyanate FITC channel (total substrate) and the Pacific Blue channels (cleaved substrate). Data were normalised to virus fusion in DMSO-treated control cells (100%, dotted line) using Microsoft Excel. Statistics were performed in GraphPad Prism using one-way analysis of variance (Anova), Fisher’s least significant difference (LSD) test.

### 2.9. Semliki Forest Virus (SFV) Cell Binding Assay

HeLa Kyoto cells (100,000 cells/well) were plated in 6-well plates in complete media. The following day, cells were treated with drugs diluted to 10 μM (or 20 μL for N-p Tosyl-L-PCK, Supercynnamaldehyde, and Raloxifene) in supplemented DMEM for 1 h at 37 °C. Next, cells were cooled on ice and infected with SFV at MOI 100 in the presence of compounds. After 1 h on ice, cells were washed and lysed (Lysis buffer: 20 mM Hepes, pH 7.4; 110 mM potassium acetate; 2 mM magnesium chloride; 0.1% Tween 20; 1% Triton X-100, 0.5% sodium deoxycholate, and 500 mM sodium chloride), and analysed by Western blot, staining with anti- SFV E1-E2 antibody [22]. Band intensities were acquired using a Licor Odyssey infrared imaging system and analysed using Image Studio Lite software.

### 2.10. SFV Internalisation Assay

HeLa Kyoto cells (100,000 cells/well) were plated in 6-well plates in 2 mL of complete media. The following day, cells were treated with drugs diluted to 10 μM (or 20 μM for N-p Tosyl-L-PCK, Supercynnamaldehyde, and Raloxifene) in supplemented DMEM for 1 h at 37 °C. Next, cells were cooled on ice and infected with SFV at MOI 100 in the presence of compounds. After 1 h on ice, the cells were moved to 37 °C to allow for viral internalisation. After 20, 40, or 60 min, the cells were cooled on ice and treated with 0.5 mg/mL subtilisin (Sigma) to remove non-internalised SFV particles. After 45 min, cells were washed extensively with cold buffer containing 1 mM AEBSF (Sigma, UK) in 0.2% bovine serum albumin/PBS, lysed, and analysed by Western blot, as described above.

### 2.11. SFV Envelope Acidification Assay

HeLa Kyoto cells (100,000 cells/well) were plated in 6-well plates in 2 mL of complete media. The following day, cells were treated with drugs diluted to 10 μM (or 20 μL for N-p Tosyl-L-PCK, Supercynnamaldehyde, and Raloxifene) in supplemented DMEM for 1 h at 37 °C. Next, cells were cooled on ice and infected with SFV at MOI 100 in the presence of compounds. After 1 h on ice, the cells were moved to 37 °C to allow for viral internalisation. After 30 min, the cells were cooled on ice, lysed in 1% Triton X100 in PBS, and treated or not with 0.8 mg/mL Trypsin (Sigma) for 10 min. Trypsin was neutralised with 2 mg/mL soybean trypsin inhibitor (Sigma) and samples analysed by Western blot, as described above.

### 2.12. SFV Lipid Mixing Assay

10 μL of 1 mM Vibrant DID (1,1′-Dioctadecyl-3,3,3′,3′-Tetramethylindodicarbocyanine) cell labelling solution (V22887, Thermo Fisher Scientific, UK) was mixed with 0.5 μL of 20% Pluronic F127 (Sigma, UK), before adding to 1 × 10^9^ pfu of SFV diluted in 1 mL of TN buffer (100 mM NaCl, 50 mM Tris, pH 8.0). The mix was vortexed at a medium speed for 5 min and then incubated at RT in the dark for 1 h. Labelled virus was separated from free dye using a 5 mL HiTrap column (GE Healthcare, UK), and the virus-containing fractions (verified by Western blot) pooled together and supplemented with 1% bovine serum albumin for storage at −80 °C. 

HeLa Kyoto cells (10,000 cells/well) were plated in Perkin Elmer ViewPlate 96-black in 100 μL complete media. The following day, cells were treated with drugs diluted to 10 μM (or 20 μL for N-p Tosyl-L-PCK, Supercynnamaldehyde, and Raloxifene) for 1 h at 37 °C. 100 nM Bafilomycin treatment was included as a positive control. Next, cells were cooled on ice and infected with 1 μL DID-labelled SFV/well in the presence of compounds. After 1 h on ice, the infectious media were removed and replaced with warmed dilutions of the same drugs. Cells were transferred at 37 °C to allow for viral internalisation. After 40 min, the cells were cooled on ice, washed, and fixed with 4% PFA. Following nuclear staining (Hoechst, 5 μL/mL), plates were imaged on a PE Opera LX, taking 25 fields/well using a 40× objective. Fluorescent puncta in the far-red channel were analysed and quantified using the PE Columbus analysis software. Analysis was performed using Microsoft Excel and GraphPad Prism. Data were normalised to lipid mixing in DMSO-treated control cells (100%, dotted line) using Microsoft Excel. Statistics were performed in GraphPad Prism using one-way Anova, Fisher’s LDS test. 

### 2.13. Exogenous Protein Expression Measurement

HeLa Kyoto cells (100,000 cells/well), plated the day before in 6-well plates, were transfected with 1 μg GL3 Renilla luciferase plasmid [23], using Fugene 6 (Promega) according to manufacturer’s instruction and a 1:3 DNA:Fugene ratio in 50 μL of Optimem (Gibco, Thermo Fisher Scientific, UK); 24 h later, the transfection medium was replaced with the indicated concentrations of compounds. Emetine was included as a positive control. Eight hours later, samples were washed once with PBS, lysed in 100 μL passive lysis buffer (Promega, UK), and following addition of Renilla Glow detection reagent (Promega, UK), luminescence was measured. Luminescence values were converted to percentages of protein expression, normalised to the DMSO-treated control. Data were normalised to protein expression in DMSO-treated control cells (100%, dotted line) using Microsoft Excel. Statistics were performed in GraphPad Prism using one-way Anova, Fisher’s LDS test.

### 2.14. Endogenous RNA Transcription Measurement

HeLa Kyoto cells (100,000 cells/well), plated the day before in 6 well plates, were treated with drugs diluted to 10 μM (or 20 μL for N-p Tosyl-L-PCK, Supercynnamaldehyde, and Raloxifene), along with Emetine-treated and untreated cells. Eight hours later, cells were lysed in 350 μL RLT buffer (RNAeasy Plus Mini Kit, Qiagen, UK) supplemented with beta-mercaptoethanol (1:100). Cells were harvested, homogenised with a G25 needle, and their RNA extracted following the manufacturer’s instructions. RNA (500 ng) was reverse-transcribed using Quantitect Reverse Transcription Kit (Qiagen). Complementary DNA (cDNA) was diluted 1:5 in deionised water and quantitative real-time polymerase chain reaction (qRT-PCR) was performed on 2 μL of cDNA using Sybr Green (BioRad, UK) to amplify the endogenous housekeeping genes actin and hypoxanthine- guanine phosphoribosyltransferase (HPRT). Samples were then run on a CFX Connect RT System using the Biorad CFX Manager program. The conditions for qRT-PCR were: 95 °C for 7 min; 40 cycles at 95 °C for 10 s, 56 °C for 30 s and 72 °C for 40 s. Data were converted to CT values and RNA expression was compared to untreated controls. Data were normalised to transcription in DMSO-treated control cells (100%, dotted line) using Microsoft Excel. Statistics were performed in GraphPad Prism using one-way Anova, Fisher’s LDS test.

### 2.15. Transferrin Internalisation

HeLa Kyoto cells (100,000 cells/well) were plated in 12 well plates and the following day compounds were added at the indicated concentrations for 40 min. Cells were next cooled on ice and 20 μg/mL Transferrin-488 (Invitrogen Molecular Probes, Alexa-Fluor 488 conjugate, T13342, Thermo Fisher Scientific, UK) was added directly into the wells for 15 min, in the presence of compounds. Next, the cells were moved to 37 °C for the indicated times to allow transferrin internalisation. At the end of the incubation, cells were washed twice with 500 μL of 0.5M Glycine, pH 2.2 for 30 s/wash, twice with PBS, and finally scraped off the plate and fixed in 4% PFA for 15 min at RT. Internalised transferrin was quantitated by flow cytometry using an LSR-II (BD) flow cytometer. Data were compared to internalisation in DMSO-treated cells; statistics were performed in GraphPad Prism using two-way Anova with Dunnett’s test for multiple comparisons.

### 2.16. In Vivo Pharmacokinetics

Pharmacokinetic studies were performed by Cyprotex on CD1 mice, as indicated in the main text.

### 2.17. Mice and Infections

AG129 mice (Marshall BioResources, Hull, UK) were maintained in pathogen-free conditions before viral infection. Infection was carried out intraperitoneally with 10^5^ FFU ZIKV (PF13/251013-18, provided by European Commission Seventh Framework Program (FP7/2007-2013) for the DENFREE project under Grant Agreement n°282 378). Mice were untreated or treated orally with Sofosbuvir (5.7 mg/kg), Monensin (10 mg/kg) or Tyrphostin A9 (1 mg/kg). Mice were pre-treated with drugs for 1 h prior to infection and then every 8 h for the first 3 days, and every 24 h for the remaining days, with the exception of Tyrphostin A9, which was only administrated four times due to toxicity. Upon infection, mice were clinically scored daily for signs of disease.

### 2.18. Zika Virus (ZIKV) RNA Quantification

ZIKV RNA was measured as previously described [24]. Briefly, RNA was isolated from plasma using the viral RNA mini kit (Qiagen, UK) and from tissues using the RNeasy mini Kit (Qiagen, UK) according to the manufacturer’s instructions. ZIKV RNA was quantified by qRT-PCR using the TaqMan RNA-to-Ct 1-step kit in a Viia 7 real-time PCR system (Thermo Fisher Scientific, UK). A standard curve using in vitro transcribed ZIKV RNA containing the complete 5’UTR of ZIKV was included to calculate the number of ZIKV RNA copies per sample. The RNA in vitro transcription step was performed using the T7 Ribomax Express Large Scale RNA Production System (Promega, UK).

### 2.19. Immunofocus Assay

Infectious ZIKV particles in mouse plasma were quantified by incubating Vero cells in a 24 well plate format (90,000 cells plated the day before) with a ten-fold serial dilution of sample. After 2 h at 37 °C, the infectious inoculum was removed and replaced with a mixture of 1.5% semisolid carboxymethylcellulose overlay (Rectapur, low viscosity, VWR, UK) in MEM. 48 h later the overlay was removed, cells washed in PBS, fixed in 4% PFA, and permeabilized for 5 min with 2% Triton X100 in PBS. After 30 min incubation in 2% milk in PBS, cells were incubated for 90 min with the 4G2 mouse anti-flavivirus antibody (MAB10216, Millipore, 1:500 dilution in 2% milk), washed 3 times in PBS, and incubated with an HRP-conjugated anti-mouse secondary antibody (Life Technologies, G21040, 1:500 dilution in 2% milk). After further washing, plaques were revealed by incubation with the HRP substrate SIGMAFAST 3-3’-Diaminobenzidine (D44418 Sigma, UK) diluted in water as per the manufacturer’s instruction and counted manually.

### 2.20. Staining of ZIKV Infected Cells and Flow Cytometry Analysis

Peripheral blood mononuclear cells (PBMCs) were cleared of red blood cells using the red blood cells lysis buffer from Alfa Aesar (Thermo Fisher Scientific, UK) according to the manufacturer’s instructions. Cells were fixed and stained using BD Cytofix/Cytoperm Fixation/Permeabilization Solution (BD, UK) and analysed using a BD Fortessa flow cytometer (BD). Flow cytometry data were analyzed using FlowJo software (Tree Star). PE-Cy7 rat anti-mouse CD11b, APC-eFluor780 rat anti-mouse CD11c, PE rat anti-mouse CD14, Pacific blue rat anti-mouse CD3, eFluor650 rat anti-mouse CD4 and APC rat anti-mouse CD8a were from BD Biosciences (UK). Anti-Flavivirus Group Antigen antibody, clone D1-4G2-4-15 was obtained from Merck (UK). The secondary Alexa Fluor 488-conjugated goat anti-mouse IgG (H+L) was from Thermo Fisher Scientific (UK).

### 2.21. Safety/Biosecurity

All animal work with infectious agents was conducted in BSL3 facilities, approved by the UK Health and Safety Executive and in accordance with local rules at Imperial College London, UK.

### 2.22. Statement on Animal Ethics

All animal work was approved by the local genetic manipulation (GM) safety committee of Imperial College London, St. Mary’s Campus (centre number GM77), and the Health and Safety Executive of the United Kingdom and carried out in accordance with the approved guidelines. All animal research described in this study was approved and carried out under a United Kingdom Home Office License, PPL 70/8932, dated 30^th^ of March 2016, in accordance with the approved guidelines, under the Animals (Scientific Procedures) Act 1986 (ASPA).

## 3. Results

### 3.1. Identification of Broad-Spectrum Antiviral Compounds

**Figure 1:**
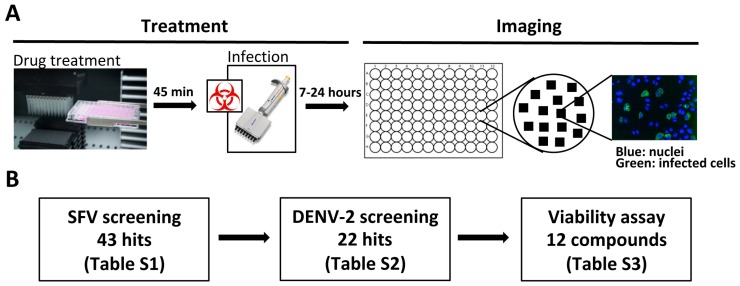
Strategy for the identification of broad-spectrum antiviral (BSA) compounds inhibiting Semliki Forest virus (SFV) and dengue virus serotype 2 (DENV-2) infection. (**A**) Schematic of the screening procedure. Cells in 96 well plates were pre-treated with 10 μM compounds for 45 min and then infected in the presence of compounds for a time sufficient to detect expression of viral proteins (7h for SFV and 24h for DENV-2). Uninfected cells, and infected cells treated with DMSO or with Monensin were included as controls. After fixation, plates were stained and images acquired using a PE Opera LX high-throughput confocal microscope, which images multiple fields in each well, allowing calculation of the percentage of infected cells. (**B**) Schematic of the screening strategy. In order to identify compounds with BSA activity, all 43 compounds displaying inhibitory activity against SFV (complete list in Table S1) were tested for their ability to block infection by DENV-2. Of the 22 compounds blocking both viruses (complete list in Table S2), the 12 least toxic, as determined by MTT assay, were selected for further studies (Table S3).

To explore whether a phenotypic screening approach could be used to identify host-targeted compounds with BSA activity, we designed the iterative screening strategy illustrated in Figure 1A,B. We initially screened ~2500 pharmacologically active (LOPAC, Sigma Aldrich) or United States Food and Drug Administration (FDA)-approved (Prestwick Chemicals) compounds for their ability to block the early stages of cellular infection by a model alphavirus, Semliki Forest virus (SFV), encoding the reporter ZsGreen [14]. HeLa Kyoto cells were pre-treated with compounds at 10 μM for 45 min, and then infected at a multiplicity of infection (MOI) of 1 (1 infectious unit per cell). Monensin, a well-known inhibitor of endosomal acidification, which has been shown to effectively block SFV entry [25], was chosen as an assay control. DMSO-treated infected and uninfected cells were also included in each plate. After 7 h, a time point that allows sufficient time for viral protein expression but precedes second rounds of infection, the inhibitory effect of each compound was assessed by comparison with DMSO controls, using high-throughput confocal microscopy. This screening identified 43 unique compounds inhibiting more than 60% of SFV infection (~1.8% hit rate; Table S1), and preserving more than 60% of cells in the wells compared to untreated controls. 

Next, in order to identify compounds with broad-spectrum activity, we tested all 43 SFV inhibitors for their ability to inhibit infection by a model flavivirus, dengue virus serotype 2 (DENV-2) (Figure 1B). Huh7 cells were pre-treated with 10 μM compounds and infected at MOI 1 for 24 h. After fixation, cells were stained to reveal the DENV E protein, and the percentage of infected cells was quantified as above. Of the 43 compounds tested, 22 inhibited DENV-2 infection by >60%, identifying molecules able to inhibit infection by members of two different viral families (Table S2).

Compounds inhibiting both viruses were re-purchased, with the exception of Emetine and Cycloheximide, two well-known inhibitors of cellular protein synthesis, and three more compounds (JS-K, Thapsigargin, and GBR 12909 dihydrochloride) that were not available at the time. With the exception of Halofantrine (IC_50_ > 100 μM), the inhibitory activity of all compounds was confirmed on SFV with IC_50_s in the high nanomolar/low micromolar range (Table S3). To limit our investigation to compounds with better selectivity indices (SI = TC_50_/IC_50_), we tested cell viability by MTT assay in HeLa Kyoto cells for 7h, and the 12 compounds with higher SI were selected for further studies (Table 1, left, and Table S3).

IC_50_ and TC_50_ values for the same 12 compounds were also determined for DENV-2 in Huh7 cells at 24 h post-infection (hpi) (Table 1, middle). As higher toxicity was observed when cells were treated for 24 h, in all assays requiring longer incubations, cells were first treated with compounds for 7 h, after which the media were replaced with compound-free media for the remaining 17 h, before perforning the MTT assay (Table 1, right). Lower toxicity was in general observed, but most compounds maintained comparable IC_50_ values. 

### 3.2. Selected Compounds Display Broad-Spectrum Activity against Other Alpha- and Flavi-Viruses

Since SFV and DENV-2 belong to two different viral families, we hypothesized that the identified compounds possess a degree of broad-spectrum inhibitory activity. To investigate whether this activity is preserved against related viruses, the compounds were tested against the alphaviruses Sindbis virus (SINV) and Ross River virus (RRV), and against the flaviviruses DENV serotypes 1 and 3 (DENV-1 and DENV-3), Zika virus (ZIKV), yellow fever 17D (YF17D), and the more distantly related member of the flaviviridae hepatitis C virus (HCV). With the exception of Raloxifene, all compounds tested were able to inhibit infection by SINV and RRV with similar potency to SFV (Table 2A). Similar levels of inhibition were also observed for DENV-1 and DENV-3. All compounds inhibited YF17D and HCV infections with similar or higher potency than DENVs while, interestingly, some compounds appeared to be less potent or not active against ZIKV. These data show that the broad-spectrum activity of most of the compounds identified in our phenotypic screening extends to other alpha- and flavi-viruses. 

### 3.3. Selected Compounds Display Broad-Spectrum Activity against Viruses from Different Viral Families

Since alpha- and flavi- viruses are known to enter cells via CME followed by pH-dependent fusion [26], we tested the activity of the same compounds against a panel of virus-like particles (VLPs) pseudotyped to mimic the entry process of vesicular stomatitis virus (VSV), rabies virus (RABV, Challenge virus standard 11), and influenza H5N1 (H5A/Vietnam/1194/2004 + N1A/PuertoRico/8/1934), which are all internalised by endocytosis and require low endosomal pH for fusion [27,28]. With the exception of Raloxifene, most compounds inhibited infection by all VLPs with similar potency to alphaviruses and flaviviruses (Table 2B). 

Differently from the viruses/VLPs tested above, the filovirus Ebola virus (EBOV) is internalised by macropinocytosis, but still fuses in late endosomes/lysosomes following pH-dependent proteolysis of its envelope glycoprotein [12]. Infection by lentiviral VLPs pseudotyped with EBOV Sudan glycoprotein was potently inhibited by most compounds. In particular, Amiodarone, Amodiaquine, Bepridil, and Raloxifene appeared to be much more potent against EBOV VLPs than against all other viruses/VLPs tested (Table 2B).

Finally, the same compounds were tested against three viruses that, in the cell system of choice, do not require CME or low pH-dependent fusion to be released into the cytoplasm: human immunodeficiency virus type 1 (HIV-1 R3A [29,30]), herpes simplex virus 1 (HSV-1 [31]) and vaccinia virus (VACV, Western Reserve [32]). Monensin did not block infection by any of these viruses, indicating pH-independence in the cell system studied. However Niclosamide, Stattic, and Tyrphostin A9 effectively inhibited replication by all three viruses, suggesting that the BSA activity of these compounds extends to viruses that use different entry and replication mechanisms. Interestingly, limited or no antiviral activity was measured for Amiodarone, Amodiaquine, Bepridil, and Raloxifene, suggesting that these compounds, which were highly potent against EBOV VLPs, are more effective against viruses that fuse in late endosomes/lysosomes (Table 2B). 

Taken together these data suggest that of the 12 compounds identified in our screen, some (including Amiodarone, Amodiaquine, and Bepridil) are likely to effectively block viruses that fuse in the endosomal compartment, while others (including Niclosamide, Stattic, and Tyrphostin A9) have an even more broad-spectrum activity, inhibiting viruses that are internalised by pH-independent plasma membrane fusion, as well as macropinocytosis and CME. A third group of drugs, including N-p-Tosyl-L-PCK, Parthenolide, Raloxifene, and Supercinnamaldehyde appear to inhibit some pH-dependent and some pH-independent viruses, and this selectivity may depend on their exact mode of action. The activity of Mitoxantrone, a cytostatic drug that intercalates into DNA forming covalent complexes [33], appears to correlate with the length of treatment, suggesting that this compound may have some irreversible toxic effect that masks the antiviral activity observed after shorter treatments. Calcimycin, an ion carrier that increases intracellular calcium levels [34], potently inhibits most of the viruses tested, but high toxicity was also observed.

### 3.4. Most Compounds Inhibit Infection before or at the Stage of Fusion

**Figure 2:**
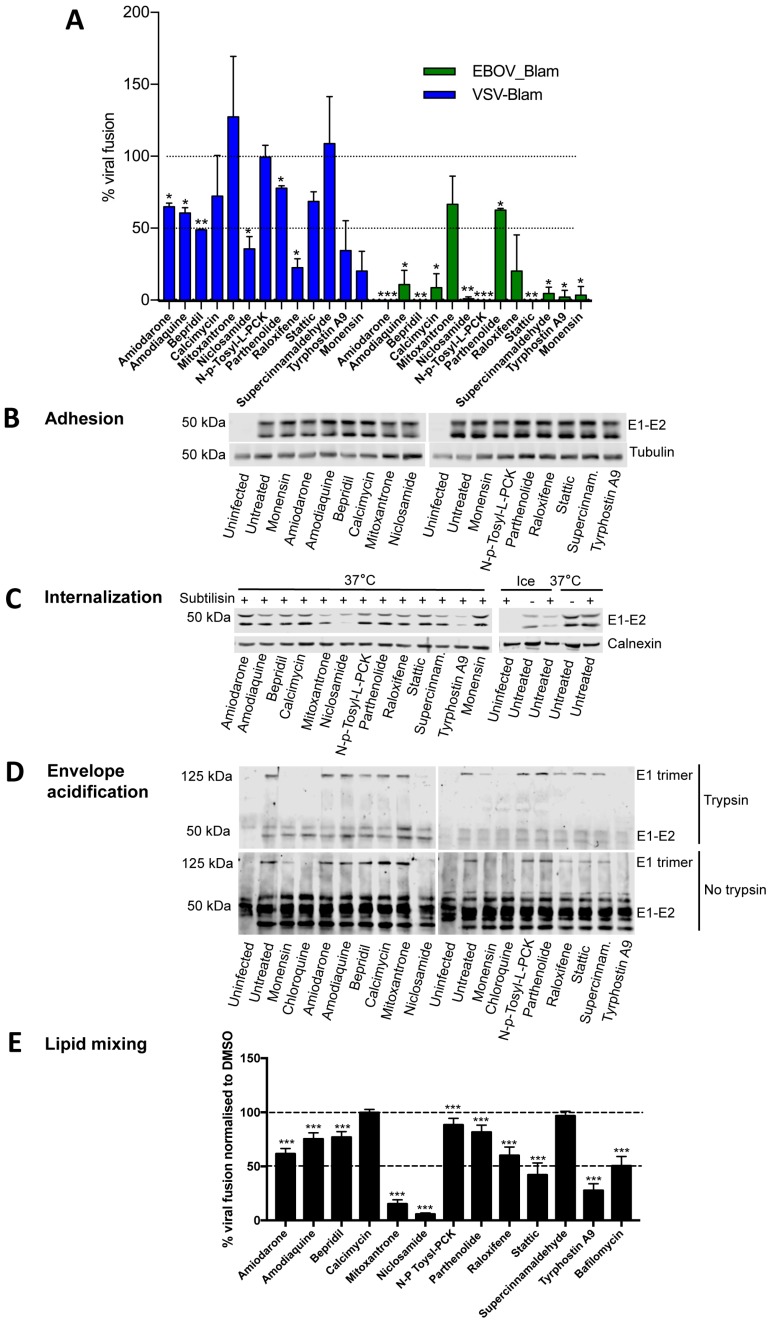
Analysis of compounds modes of action in the virus entry pathway. (**A**) Percentage of VSV_Blam (blue) or EBOV_Blam (green) pseudotyped VLP fusion events upon compound treatment, measured as cytosolic release of β-lactamase. HeLa Kyoto cells were treated with the indicated compounds for 1 h before infection with pseudotyped Blam VLPs. After 1 h at 37 °C, compounds were removed, the β-lactamase substrate added, and the percentage of cells in which fusion had occurred was quantified by flow cytometry. Data are normalised to Blam signal in DMSO-treated control cells (100%, dotted line). Monensin was used as a positive control. Statistics: one-way analysis of variance (Anova), Fisher’s least significant difference (LSD) test. * = *p* < 0.05; ** = 0.01 > *p* > 0.005; *** = *p* < 0.005. (**B**) Western blots showing the amount of SFV E1/E2 proteins that remains bound to the surface of infected HeLa Kyoto cells after 1 h compound treatment at 37 °C, and 1 h SFV infection on ice, in the presence of compounds. Untreated samples were included as controls. A Western blot for tubulin was used as a loading control. (**C**) Western blots showing SFV E1/E2 protein after subtilisin treatment. HeLa Kyoto cells were treated with the indicated compounds for 1 h at 37 °C, and SFV bound for 1 h on ice in the presence of compounds. Next, virus was allowed to internalise at 37 °C for 20 min, before subtilisin treatment on ice to remove surface-bound virus Ice-treated samples (where the virus was not internalised) treated or not with subtilisin, as well as untreated samples incubated at 37 °C (where the virus was internalised) were included as controls. (**D**) Western blot showing SFV E1/E2 proteins and low pH-induced E1 trimers. HeLa Kyoto cells were treated with the indicated compounds for 1 h at 37 °C, SFV bound 1 h on ice in the presence of compounds, and then internalised at 37 °C for 40 min, before cell lysis. A fraction of each lysate was treated trypsin to verify the identity of the trypsin-resistant E1 trimer (top panel). Monensin and Chloroquine (100 μM), known inhibitors of endosomal acidification were used as positive controls. Untreated samples were included as negative controls. (**E**) Percentage of DID-labelled SFV hemifusion/fusion events normalised to DMSO treated cells (100%, dashed line). HeLa Kyoto cells were pre-treated with compounds for 1 h at 37 °C before adding DID-SFV for an additional hour on ice. Unbound virus was then washed away and infection left to proceed for 40 min at 37 °C to allow virus internalisation and fusion. Bafilomycin (100 nM), a known inhibitor of viral fusion, was used as positive control. Hemifusion/fusion events were quantified on a PE Opera LX. Averages from three independent experiments are shown. Statistics: one-way Anova, Fisher’s LSD test. * = *p* < 0.05; ** = 0.01> *p* > 0.005; *** = *p* < 0.005.

Since the readout of the infectivity assays described so far is expression of viral protein, each drug could potentially inhibit any stage from virus binding to the cell surface through to viral protein synthesis. In order to identify inhibitors of the viral entry process, i.e., prior to RNA or protein synthesis, we tested the ability of each compound to prevent cytosolic release of β-lactamase (Blam) from inside VLPs pseudotyped with VSV-G or EBOV glycoprotein (Figure 2A). Similarly to Monensin, Niclosamide, Raloxifene, and Tyrphostin A9 inhibited more than 50% of VSV_Blam_VLP fusion, compared to DMSO controls. Amiodarone, Amodiaquine, Bepridil, Calcimycin, Parhenolide, and Stattic also appeared to inhibit VSV_Blam_VLP, although to a lesser extent. With the exception of Mitoxantrone, all compounds also blocked EBOV_Blam_VLP fusion, most of them more effectively than VSV_Blam_VLP, consistent with the IC_50_ data (Table 2B). Together these data suggest that whilst most of the compounds identified are able to inhibit during or before the stage of virus fusion to some extent, their efficacy is likely to depend on the site of viral fusion, with viruses fusing in the late endosomes/lysosome like EBOV inhibited more effectively than viruses fusing in the early endosome, like VSV or SFV.

To further characterise the inhibitory mechanisms of each compound on entry steps upstream of viral fusion, we investigated their ability to block different stages of SFV entry. Upon binding to the cell surface, SFV is internalised by CME and traffics to the early endosomes. Here, the acidic pH triggers conformational changes in the envelope protein E1 that allows fusion between the viral envelope and the endosomal membrane, leading to release of the SFV genome into the cytoplasm [35]. 

First, we tested inhibition of SFV adhesion to the cell surface. Cells were pre-treated with drugs for 1 h, and then incubated with virus at high MOI (100) for an additional hour on ice to allow virus adhesion to the cell surface, but no internalisation. Cells were then lysed and the amount of virus bound to the cell surface analysed by Western blotting, measuring the SFV envelope proteins E1/E2. No significant differences were observed between treated and untreated control samples, suggesting that none of the compounds prevents virus attachment to the cell surface (Figure 2B; quantification in Figure S1A).

Next we tested whether drug treatment caused inhibition of virus endocytosis. Pre-treated cells were allowed to bind SFV at MOI 100 for 1 h on ice, and then transferred to 37 °C for 20 min to allow virus internalisation prior to subtilisin treatment on ice, which removes virus remaining at the cell surface. Internalised virus was quantitated by Western blotting for the viral envelope proteins. Complete loss of E1/E2 was observed in the control samples held on ice (where virus internalisation does not occur), but not in untreated controls incubated at 37 °C for 20 min (where virus is internalised). Loss of E1/E2 was also observed after treatment with Niclosamide or Tyrphostin A9, suggesting that these two compounds are able to prevent virus internalisation (Figure 2C; quantification in Figure S1B).

Next, we tested whether any of the compounds inhibit endosomal acidification, preventing the conformational changes in the SFV envelope glycoproteins required for fusion. Upon endosomal acidification, SFV E1 rearranges into trypsin resistant trimers [36]. Pre-treated cells were infected with SFV at MOI 100 for 1 h on ice to synchronise infection and then transferred to 37 °C for 30 min to allow virus internalisation and fusion. Cells were then lysed and treated with trypsin to assess trimer formation and to confirm its trypsin resistance. As expected, Monensin and Chloroquine, which inhibit endosomal acidification, both prevented trimer formation (Figure 2D; quantification in Figure S1C). No trimers were observed in the presence of Niclosamide and Tyrphostin A9, possibly a consequence of their upstream inhibition of virus internalisation. No other compound appeared to significantly inhibit E1 trimer formation compared to untreated controls, suggesting that their inhibitory effect is likely to occur after endosomal acidification. 

Finally, we tested whether any of the selected compounds affect hemifusion (lipid mixing between the external leaflets of the viral and endosomal membranes) by quantifying the de-quenching of DID-labelled SFV. DID is a lipophilic tracer that self-quenches when incorporated at high concentrations into viral membranes, but de-quenches following hemifusion or full fusion [37,38]. Lower signal than in the DMSO control is indicative of inhibited hemifusion/fusion. Pre-treated cells were infected with SFV-DID at MOI ~100 for 1 h on ice, and then for 40 min at 37 °C to allow fusion. De-quenching was visualised and quantified on a high-content confocal microscope and normalised to DMSO controls (Figure 2E). Strong inhibition of de-quenching was observed in samples treated with Bafilomycin, an inhibitor of endosomal acidification and viral fusion [39], as well as in samples treated with Niclosamide and Tyrphostin A9, most likely as a consequence of inhibited entry. More than 50% inhibition was also observed for Mitoxantrone and Stattic, although an unusual staining pattern for Mitoxantrone suggested a possible artefact. Intermediate levels of inhibition were observed for Amiodarone, Amodiaquine, Bepridil, and Raloxifene, suggesting that these compounds inhibit hemifusion to some extent. With the exception of Mitoxantrone, these data are broadly in agreement with the β-lactamase fusion assay (Figure 2A), suggesting that compounds that block β-lactamase delivery into the cytoplasm most likely do so by preventing membrane fusion, with the possible exception of Niclosamide and Tyrphostin A9 that block virus internalisation.

**Figure 3:**
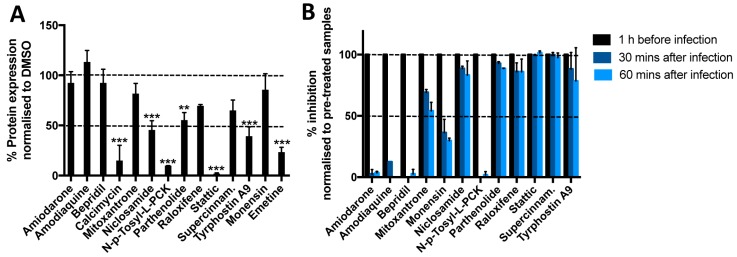
Analysis of compounds mode of action after fusion. (**A**) A Renilla expressing plasmid was transfected in HeLa Kyoto cells. 24 h later, cells were treated with compounds for 8 h and then lysed to measure Renilla activity. Data are normalised to a DMSO treated control (100%). Emetine (10 μM), a known inhibitor of protein synthesis, was used as a control. Averages of three independent experiments are shown. Statistics: one-way Anova, Fisher’s LSD test. * = *p* < 0.05; ** = 0.01> *p* >0.005; *** = *p* < 0.005. (**B**) Percentage of inhibition of SFV infection following administration of each compound 60 min before infection, or at 30 and 60 min after infection. Cells (HeLa Kyoto) were fixed at 7 hpi and percentages of infection quantified following images acquisition on a PE Opera LX. Data are normalised to pre-treated controls samples (100% inhibition). Averages of two independent experiments are shown. Statistical significance is shown in Table S4, as determined with a two-way Anova with Dunnett’s test for multiple comparisons.

Following capsid release, the SFV genome is immediately translated and then replicated. To test whether any of the compounds inhibited protein synthesis (whether due to toxicity or a more specific mechanism), we transfected HeLa Kyoto cells with a Renilla luciferase construct, and 24 h later treated cells with compounds for 8 h. Emetine, a potent inhibitor of protein synthesis, was included as control and significantly reduced the Renilla signal compared to untreated controls (Figure 3A). A decrease of more than 50% was measured following treatment with Calcimycin, Niclosamide, N-p-Tosyl-L-PCK, Stattic, and Tyrphostin A9. At lower concentrations (below their IC_50_ against SFV) Calcymicin and Stattic both lose their inhibitory activity on protein synthesis, suggesting that their antiviral activity is not entirely due to reduced protein synthesis (Figure S1D). At lower concentrations N-p-Tosyl-L-PCK partially inhibits protein synthesis but not virus infection, while Niclosamide and Tyrphostin A9 show similar levels of inhibition of protein synthesis and viral replication at all three concentrations tested, suggesting that the inhibitory activity of these compounds is likely to be due to inhibition of both virus entry and translation. Inhibition of protein synthesis is not a direct consequence of inhibition of transcription, as none of the drugs appeared to significantly reduce transcription of cellular mRNA (Figure S1E). 

To further distinguish between the effects of each compound on pre- vs. post-fusion stages of infection, we performed time-of-addition assays where compounds were added either before SFV, or 30 and 60 min after synchronous internalisation (Figure 3B, statistics in Table S4). No difference was observed for Stattic and Supercynnamaldehyde when the drugs were added before infection or 30 and 60 min after, suggesting that these two drugs are likely to act after viral fusion. A minor but reproducible decrease in activity was observed when Niclosamide, Parthenolide, Raloxifene, and Tyrphostin A9 were added at later time points, suggesting that this group of drugs is likely to act both before and after fusion. A progressive and more dramatic decrease in activity was observed for Mitoxantrone and Monensin, suggesting that these drugs primarily act during or before the fusion process, but may have some residual activity after fusion. Finally, Amiodarone, Amodiaquine, Bepridil, and N-p-Tosyl-L-PCK appear to act exclusively before (or at the stage of) fusion. These data are broadly in agreement with the fusion assays (Figure 2A,E). All four drugs displaying very broad antiviral activity (Niclosamide, Calcimycin, Stattic, and Tyrphostin A9) appear to act both before and after fusion, while drugs blocking exclusively pH-dependent viruses (Amiodarone, Amodiaquine, and Bepridil) inhibit primarily pre-fusion/fusion stages. A summary of the antiviral activities of all compounds and of the stages of viral infection on which they appear to act is summarised in Table 3. Together these experiments suggest that Amiodarone, Amodiaquine, Bepridil, and Raloxifene act by inhibiting viral fusion, while Niclosamide and Tyrphostin A9 inhibit both viral internalisation and translation. 

### 3.5. Niclosamide and Tyrphostin A9 Inhibit Viral Entry by Reducing Clathrin-Mediated Endocytosis

**Figure 4:**
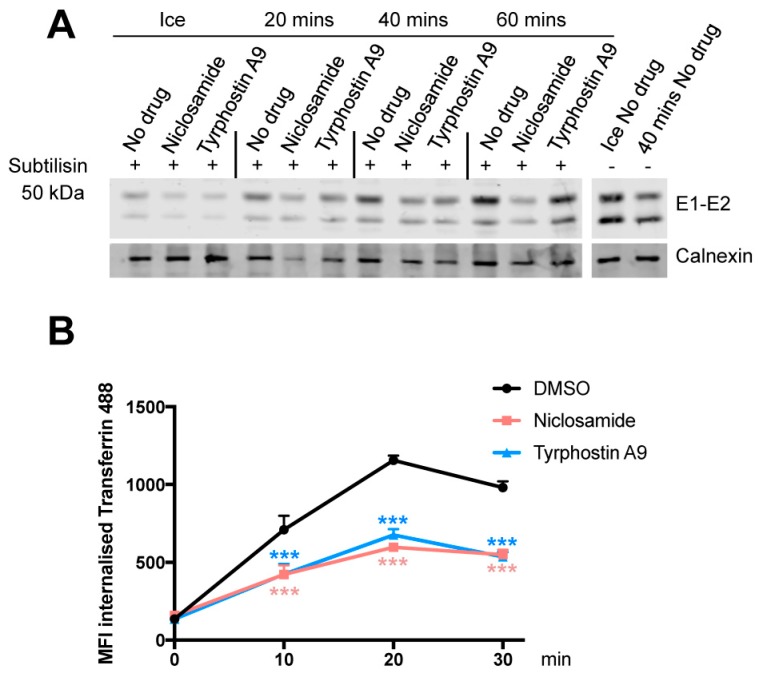
Niclosamide and Tyrphostin A9 impact on endocytosis. (**A**) Western blot showing SFV E1/E2 protein after subtilisin treatment. HeLa Kyoto cells were treated with 10 μM Niclosamide or Tyrphostin A9 for 1 h at 37 °C, and infected with SFV for 1 h on ice in the presence of compounds. Virus was left to internalise at 37 °C for 20, 40, or 60 min, before subtilisin treatment to remove surface-bound virus. Untreated cells not exposed to subtilisin, were included as controls. (**B**) HeLa Kyoto cells were treated with 10 μM Niclosamide or Tyrphostin A9 for 1 h before internalisation of Alexa 488-conjugated transferrin for 10, 20, and 30 min. DMSO-treated cells were used as controls. One representative of three independent experiments is shown. Statistics: two-way Anova with Dunnett’s test for multiple comparisons. *** = *p* < 0.005.

To further clarify the mechanism behind Niclosamide and Tyrphostin A9 inhibition of SFV entry, we investigated the kinetics of virus internalisation by measuring resistance to subtilisin treatment. While slightly higher levels of internalisation were observed after 60 min at 37 °C, particularly for Tyrphostin A9, the amount of internalised virus remained lower compared to untreated control at all other time points, suggesting that internalisation is delayed or inhibited over time (Figure 4A).

Next we tested whether these compounds also inhibit the uptake of a normal endocytic cargo, specifically the clathrin-mediated uptake of transferrin. Using fluorescent transferrin-488, we saw that both drugs reduced transferrin internalisation at each time point tested compared to DMSO controls (Figure 4B), suggesting that the inhibitory activity of these compounds is not specific to virus particles, but a more general inhibition of CME.

### 3.6. Tyrphostin A9 and Monensin Reduce Viral Titres in the Blood and in the Lymph Nodes

While entry inhibitors can be effective antivirals in vitro, very little information is available on their activity in vivo. We therefore tested whether the antiviral activity of some of the compounds identified in our screen was maintained in an in vivo model of infection, providing proof-of-concept for our host-targeted phenotypic approach. Of the 12 compounds identified in the screen, we decided to test Niclosamide and Tyrphostin A9 in vivo, as these were the most potent compounds in the in vitro assays, and at least part of their activity appears to be due to inhibition of CME.

Effectiveness in vivo also depends on the bio-distribution of the compound, which needs to be present at appropriate concentration at sites where virus replicates/circulates. Oral dosage (PO) of Niclosamide delivers the drug to the intestinal lumen where it exerts its known antihelminthic activity. However its poor solubility both in water and oil limits absorption into the blood following oral administration [40]. Studies in rats showed that one third of the orally dosed Niclosamide is absorbed from the gastrointestinal tract and is eliminated in the urine within 24 h, while two thirds is eliminated in the faeces [41]. Intraperitoneal (IP) administration of 10 mg/kg of Niclosamide in CD1 mice resulted in a peak blood concentration of 40 μM 15 min after injection, but undetectable levels after 2 h (Figure S2A). Given this poor bioavailability we decided not to test Niclosamide further.

Because of toxicity at 10 mg/kg, Tyrphostin A9 was dosed at 1 mg/kg. PO dosage results in a peak blood concentration of 1.52 μM 1 h after administration, and 0.76 μM after 8 h (Figure S2B). IP dosage results in a blood peak of 5 μM, but only 20 nM concentrations after 2 h (Figure S2C). PO dosage of Tyrphostin A9 was therefore selected for antiviral testing against ZIKV in interferon receptors I and II deficient AG129 mice, a well established model of flavivirus infection [42,43].

**Figure 5:**
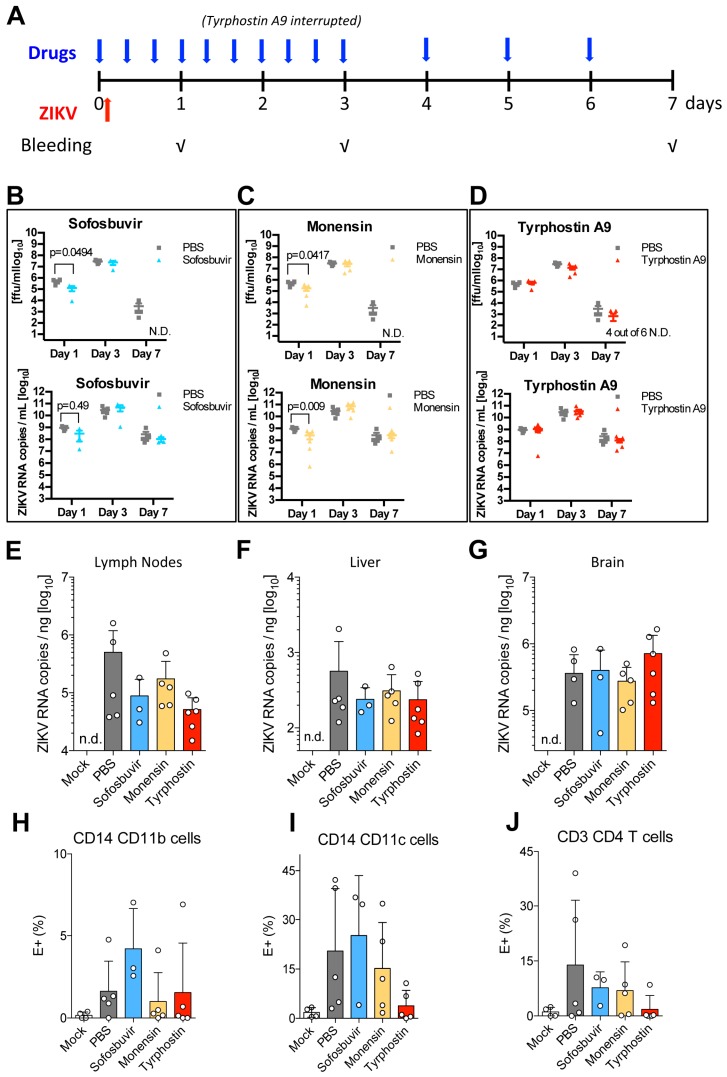
In vivo activity of Tyrphostin A9, Monensin, and Sofosbuvir. (**A**) Schematic of the drug treatment and infection regime in AG129 mice. Mice were treated with 5.7 mg/kg of Sofosbuvir, 10 mg/kg of Monensin, or 1mg/kg of Tyrphostin A9 before and after infection with 10^5^ pfu of ZIKV, at the indicated intervals. Administration of Tyrphostin A9 was suspended at day 3 due to toxicity. Focus-forming units (ffu, top panels) and ZIKV RNA copies (bottom panels) per ml of serum at day 1, 3, and 7 p.i. upon treatment with Sofosbuvir (**B**), Monensin (**C**), or Tyrphostin A9 (**D**). N.D. (not detected), indicates that no infectious units were recovered. ZIKV RNA copies/ng at day 7 p.i. in the lymph nodes (**E**), liver (**F**), and brain (**G**) upon indicated treatment. Percentages of CD14^+^CD11b^+^ macrophages (**H**), CD14^+^CD11c^+^ DC (**I**), and CD3^+^CD4^+^ T cells (**J**) infected with ZIKV at day 7 p.i. upon indicated treatment. P values from unpaired T tests are displayed for statistically significant comparisons.

The antiviral activity of Monensin in vivo was also tested at a concentration of 10 mg/kg. Monensin is toxic in humans, but is well tolerated in mice at concentrations up to 70 mg/kg [44]. Sofosbuvir, a potent inhibitor of HCV [45] with some activity against ZIKV in vitro [46] and in vivo [47], was also included at 5.7 mg/kg. All drugs where administered orally 1 h before infection, then every 8 h for the first 3 days, and every 24 h for the remaining days. Tyrphostin A9 administration was suspended after the fourth administration because of toxicity and weight loss (Figure 5A). Blood was collected at day 1, 3, and 7 post infection (p.i.) 

ZIKV infection of AG129 mice has been reported to be lethal at 7–8 days p.i. even at infectious doses as low as 1 pfu/mL [42], suggesting that only antivirals that completely clear the virus would prevent lethality. As expected from this severely immune compromised model, due to severe weight loss and signs of illness, all mice were culled at day 7, independently of the administered treatment, which did not prevent body weight loss relative to untreated ZIKV-infected mice (Figure S3A). 

Analysis of ZIKV titres in the blood at different time points revealed differences in compound-treated vs. non-treated animals. ZIKV presence in the blood was measured both by qRT-PCR, which quantifies viral RNA, and by immunofocus assay, which quantifies infectious virus. Consistent with results reported in the literature [42], we observed the highest viraemia at day 3 p.i. and almost complete clearance at day 7 p.i. Sofosbuvir and Monensin significantly reduced blood titres at day 1 p.i, as determined both by qRT-PCR and immunofocus assay, and cleared infectious virus from the blood by day 7. Conversely, infectious virus particles were still detected in untreated samples. While only minor and non significant differences where observed at days 1 and 3 in Tyrphostin A9 treated samples, by day 7 p.i. infectious virus was detected in only two of six mice, suggesting that this group also cleared virus from the blood slightly more rapidly than untreated controls (Figure 5B–D). 

Consistent with lower viral titres in the blood in all compound-treated samples at day 7 p.i., fewer viral genome copies were detected in the lymph nodes of mice treated with all compounds, although the differences did not reach significance (Figure 5E). Fewer genome copies were also measured in the liver (Figure 5F), however, much lower levels of viral RNA were detected in this organ compared to brain and lymph nodes, regardless of treatment (Figure S3B), and no infectious virus was recovered by immunofocus assay. No differences in ZIKV titres were seen in the brain (Figure 5G), but it should be noted that the ability of these compounds to pass the blood–brain barrier is unknown. 

When we examined which blood cell populations where infected with ZIKV and whether any compound reduced the number of infected cells in the blood by immunostaining, we observed very limited/no infection of CD8+ T cells (Figure S3C) and detectable infection of CD14+CD11b+ macrophages, CD14+CD11c+ dendritic cells, and CD4+ T cells (Figure 5H–J). Interestingly, we saw a trend towards fewer infected CD4+ T cells upon treatment with all drugs (Figure 5J), and fewer infected CD14+CD11c+ dendritic cells upon Tyrophostin A9 treatment (Figure 5I), although without statistical significance. 

## 4. Discussion

In recent years, frequent viral outbreaks have highlighted the need for new therapeutic measures that may raise preparedness against both known and unknown viruses. Given the time required to develop vaccines or to move from a candidate compound to an approved drug, compounds with broad-spectrum activity against different viral agents that could be readily deployed during an outbreak would be invaluable.

This work provides proof-of-concept that inhibition of highly conserved cellular pathways, in particular pathways involved in viral entry, is a viable broad-spectrum antiviral strategy. A two-step phenotypic screen, first against an alphavirus and then against a flavivirus, identified compounds able to inhibit replication of not only other viruses from the same genera, but also viruses from different orders, families, and genera that use similar entry mechanisms, and in some instances viruses for which different entry pathways have been proposed. This provides a strong framework to develop antiviral compounds active against multiple viral families, with potential application against emerging viruses. The use of a phenotypic screening approach in this context is extremely powerful, as it allows the probing of pharmacological space in an unbiased manner, potentially revealing novel mechanisms and targets, even within well-established pathways. 

We also developed and described several assays and tools to determine the stage/s of the viral life cycle that are inhibited by each compound, thereby providing an indication of their mode of action, at least for SFV. This is important information in drug development and gives confidence on the specificity of the approach. As indicated by DID labelling experiments, most of the compounds identified in our screens appear to inhibit SFV fusion after acidification of the viral envelope, most likely at the stage of lipid mixing between viral and endosomal membrane. Some of these compounds have already been described as antivirals [48,49,50], and some, e.g., Amiodarone, have been tested in patients with questionable efficacy [51,52]. However, while inhibition of fusion may be a potentially valid antiviral strategy, the concentrations of compounds required to have an effect in vivo is likely to be high. We also noticed that these compounds are more effective against viruses that fuse late in the endocytic pathway, suggesting that their accumulation may be pH-dependent and that they would, therefore, be less effective against viruses that penetrate from early endosomes.

The two most potent and broad-spectrum compounds identified in our screen were Niclosamide and Tyrphostin A9, which appeared to inhibit both virus and transferrin internalisation. This suggests that both compounds inhibit endocytosis. Indeed, Tyrphostin A9 may possibly inhibit the interaction between tyrosine-containing endocytic signals in cargo-receptor proteins (such as the transferrin receptor) and the medium chain (μ2) subunit of the AP-2 complex, as reported for Tyrphostin A23 [53]. Niclosamide inhibition of certain alpha- and flaviviruses, including DENV, ZIKV, and chikungunya virus has been reported by others [54,55,56,57]. While different modes of action have been suggested in these studies, Niclosamide has also been shown to be a proton carrier that prevents endosomal acidification [56,58]. It is possible that Niclosmide inhibition of endocytosis is due to this increase in endosomal pH which may in turn impair endosomal trafficking. Both Niclosamide and Tyrphostine A9 also appear to have multiple mechanisms of action, including inhibition of ectopic protein synthesis, which may explain their effectiveness and potency against viruses that do not use CME as their main route of entry. It is possible that some of these mechanisms are due to the ability of Niclosamide to interfere with ATP synthesis via mitochondria uncoupling [59]. Since a similar activity has been reported for Tyrphostin A23, it is possible that also Tyrphostin A9 may interfere with ATP synthesis as well as with endosomal acidification [59]. While Tyrphostin A9 is unlikely to be a good drug candidate due to its likely interaction with multiple tyrosine kinases and the strong toxicity that we have observed in vivo, Niclosamide is an FDA-approved drug with good safety profile. Unfortunately, the pharmacokinetics of Niclosamide when dosed orally or IP indicate that it fails to accumulate in the blood or in sites important for ZIKV replication, consequently we chose not to test it further in vivo. Whilst further drug design is required to identify an analogue with improved biodistribution, there is the possibility that Niclosamide, which accumulates in the intestine, may have antiviral properties against gastro-intestinal viruses such as noroviruses or enteroviruses. Intriguingly, Niclosamide antiviral activity has been described for other pH-dependent viruses [54,55,56,57], including enteroviruses [58]. While the combination of antiviral activity and pharmacokinetics studies on Tyrphostin A9 suggested sufficient levels of exposure compared to Niclosamide, we have no indication of where this compound accumulates and whether it reaches sites of viral replication such as the brain. 

While a few inhibitors of endocytosis are already know for their antiviral activity, they mostly remain tool compounds untested in vitro [60]. Importantly, we also showed that compounds identified by phenotypic screening can reduce viral titres in vivo. While ZIKV infection of interferon receptor-deficient mice is lethal (infection of immunocompetent mice is not pathogenic), and mice had to be culled before being able to see any recovery, lower viral titres were measured in blood and lymph nodes of compound-treated mice at different time points. Although the inhibitory effect was modest and further studies would be necessary to understand the effect of any decrease in viral titres on pathogenesis, these results help link in vitro phenotypic data with in vivo efficacy. Given the host-targeted and broad-spectrum nature of our approach, we would expect an effective drug to lower (rather than completely clear) viral load, down to levels where an infection might be more readily controlled by host immune responses, or where pathogenesis is attenuated. Ample evidence exists that high viral load is associated with disease severity and often with an exacerbated immune response [61,62], and that rapid control of viral replication early in infection can dramatically change the clinical outcome [63]. While we could not test this hypothesis in the severely immune-compromised AG129 model, our results in vivo are proof of concept in support of larger phenotypic screens or targeted approaches. 

The increased risk of toxicity compared to virus-targeting drugs is the main objection to a host-targeted strategy. Aiming to lower rather than abolish viral load may allow dosing to be reduced to a level where fundamental cellular processes still operate, but virus replication is compromised. The fine balance between inhibiting viral replication and limiting toxicity will have to be determined empirically in vivo for each drug. We also anticipate that drug exposure would be limited in time for viruses causing acute infections, reducing the risk of long-term side effects. 

While we show that it is possible to identify compounds with broad-spectrum antiviral activity in vitro, it is likely that a broad-spectrum formulation will contain a cocktail of compounds to expand coverage against different viral agents and different cellular pathways. A combination of drugs targeting different cellular components may also allow the dosage of each individual compound to be lowered, thereby limiting toxicity and side effects. Indeed, our studies suggest that simultaneously blocking multiple cellular pathways important for viral replication (e.g., entry and protein synthesis) may be the most effective antiviral strategy. This calls for further studies on fundamental mechanisms of cell-virus interaction to help the design of a wider range of broad-spectrum drugs and formulations. Also, the identification of compounds with antiviral activity from the screening of just a small collection of molecules, justifies screening larger sets and novel compounds in order to identify more potent compounds with lower toxicity and suitable bio-distribution that could be developed into antiviral drugs.

## 5. Conclusions

This work provides proof-of-concept that compounds which inhibit key cellular pathways required for the early stages of entry and replication of multiple viral groups can have broad-spectrum antiviral activity in vitro, and have the potential to reduce viral replication in vivo. Iterative phenotypic screens followed by a molecular characterisation of the mode of action of the most interesting hit compounds constitute an effective strategy to identify host-targeted broad-spectrum antivirals.

## Figures and Tables

**Table 1:** IC_50_, TC_50_ and Selectivity Index (SI, equal to TC_50_/IC_50_) for the indicated compounds against SFV (in Hela Kyoto cells) and DENV-2 (in Huh7 cells). Cells were pre-treated for 45 min with serial dilutions of each compound and then infected with SFV in the presence of compounds for the duration of the experiment (7 h). DENV-2 infected cells were treated with serial dilutions of compounds for the entire duration of the experiment (24 h) or for 7 h before media replacement. After fixation, plates were stained; images acquired using a PE Opera LX were quantified to determine percentages of infection. MTT assays matched the duration of the infection, regardless of the duration of the drug treatment. A “~” symbol indicates that the non-linear regression curve fit is ambiguous, as defined by the GraphPad Prism software, i.e., the curve does not accurately accommodate the values of all the parameters. The IC_50_ and TC_50_ values derive from three independent experiments.

	SFV (7 h) (µM)			DENV-2 (24 h) (µM)			DENV-2 (7 + 17 h ) (µM)		
	IC_50_	TC_50_	SI	IC_50_	TC_50_	SI	IC_50_	TC_50_	SI
**Amiodarone**	9	96.8	10.8	12.7	~25.5	2.0	15.5	40.6	2.6
**Amodiaquine**	8.5	>50	>5.8	17.2	18.4	1.1	12.7	44.2	3.5
**Bepridil**	9.5	34.9	3.7	12.1	~23.4	1.9	15.16	~48.8	3.2
**Calcimycin**	0.6	4.1	6.8	0.38	6.7	17.6	4.4	>50	>11.3
**Mitoxantrone**	7.0	43.2	6.2	2.1	4.6	2.2	2.0	7.9	4.0
***Monensin (cntrl)***	*1.1*	*>50*	*>45*	*3.8*	*>50*	*13.2*	*4.5*	*>50*	*>11.1*
**Niclosamide**	3	>50	>16.7	0.4	12.8	32.0	1.1	>50	>45.5
**N-p-Tosyl-L-PKC**	10.7	42.7	4.0	7.5	31.7	4.2	19.6	24.2	1.2
**Parthenolide**	3.3	11.7	3.5	7.5	11.9	1.6	12.2	35.8	2.9
**Raloxifene**	15.1	>50	>3.3	9.8	~22.63	2.3	5.7	24.8	4.4
**Stattic**	3.7	7.8	2.1	0.9	9.7	10.8	3	17.3	5.8
**Supercinnamaldehyde**	15.8	40.5	2.6	15.0	25.8	1.7	15.4	59.6	3.9
**Tyrphostin A9**	2.9	>50	>17.2	0.4	28.5	71.2	1.2	>50	>41.7

**Table:** **A**

	SINV (µM)	RRV (µM)	DENV-1 (µM)	DENV-3 (µM)	ZIKV (µM)	YF17D (µM)	HCV (µM)
**Amiodarone**	~11.8	3.2	~20	~25	No inhibition	8.12	3.9
**Amodiaquine**	11.3	6.2	~24.8	~25	No inhibition	5.3	4.2
**Bepridil**	14.6	4.6	~20	~20	~25.2	7.1	~12.4
**Calcimycin**	2.1	6.5	4.3	NA	17.1	0.4	1.45
**Mitoxantrone**	9.9	4.9	5.6	6.3	~25	2.2	2.7
**Monensin (cntrl)**	0.4	4.6	6.2	~3.6	5.5	< 0.3	2.5
**Niclosamide**	2.8	0.82	1.5	1.6	~0.7	< 0.3	0.2
**N-p-Tosyl-L-PCK**	11.9	~6.3	25.6	24.2	24.3	4.6	~13.0
**Parthenolide**	3.2	7.7	10	15.8	6.1	6.4	5.6
**Raloxifene**	30.6	ND	8.8	5.3	ND	7.8	3.9
**Stattic**	3.6	3.8	3	NA	4	4.9	3.7
**Supercinnamaldehyde**	24.9	18.9	~22.7	12.2	No inhibition	7.6	15.8
**Tyrphostin A9**	~0.8	0.6	1.4	1.2	<0.3	< 0.3	<0.3

**Table:** **B**

	VSV	RABV	H5N1	EBOV	HIV	HSV (Vero)	VACV
**System**	VLP	VLP	VLP	VLP	virus	virus	virus
**Family**	Rhabdov.	Rhabdov.	Orthomyxov.	Filov.	Retrov.	Herpesv.	Poxv.
**Time of assay**	24 h	24 h	48 h	48 h	24 h	8 h	8 h
**Entry mechanism**	CME	CME	CME	Macrop.	PM fusion (?)	PM fusion	Macrop.
**Fusion**	EE	EE	LE	LE	PM (?)	PM	EE
**low pH dependency**	Yes	Yes	Yes	Yes	No	No	No
**Amiodarone**	25	No inhibition	12.5	0.9	No inhibition	24.6	No inhibition
**Amodiaquine**	11.2	23.5	13.6	0.9	No inhibition	28.22	No inhibition
**Bepridil**	10.4	6.9	8.2	0.8	No inhibition	No inhibition	26.8
**Calcimycin**	0.1	0.7	0.4	0.5	2.5	<0.5	NA
**Mitoxantrone**	0.6	<0.5	2.8	0.1	NA	6.8	No inhibition
**Monensin (cntrl)**	0.7	0.8	0.9	4.5	No inhibition	No inhibition	No inhibition
**Niclosamide**	3.5	1.3	1.4	2.9	3.3	1.3	3
**N-p-Tosyl-L-PCK**	11.5	15	No inhibition	No inhibition	12.5	No inhibition	14.4
**Parthenolide**	2.6	3.8	19.4	Toxic	3.7	No inhibition	5
**Raloxifene**	12.7	No inhibition	No inhibition	1.4	No inhibition	23.5	20
**Stattic**	3.0	3.2	Toxic	Toxic	3.2	5.2	4.1
**Supercinnam.**	14.6	21.6	17.5	21.2	No inhibition	25.2	No inhibition
**Tyrphostin A9**	4.1	1.4	<0.5	3.2	1.1	1.5	3.4

**Table:** 

	Adhesion	Internal.	E1 Acid.	Translation	Time of Addition	Hemifusion	Fusion		Viruses Inhibited
							VSV_Blam	EBOV_Blam	
**Amiodarone**	X	X	X	X	Before fusion	V	V	V	Endosomal pH-dep.
**Amodiaquine**	X	X	X	X	Before fusion	V	V	V	Endosomal pH-dep.
**Bepridil**	X	X	X	X	Before fusion	V	V	V	Endosomal pH-dep.
**Calcimycin**	X	X	X	V	After fusion	X	X	V	All tested
**Mitoxantrone**	X	X	X	X	Before and after fusion	ND	X	X	Unclear pattern
**Niclosamide**	X	V	V	V	Before and after fusion	V	V	V	All tested
**N-p-Tosyl-L-PCK**	X	X	X	V	Before fusion	X	X	V	Unclear pattern
**Parthenolide**	X	X	X	V	Before and after fusion	X	X	V	Unclear pattern
**Raloxifene**	X	X	X	X	Before and after fusion	V	V	V	Unclear pattern
**Stattic**	X	X	X	V	After fusion	V	X	V	All tested
**Supercinnamaldehyde**	X	X	X	X	After fusion	X	X	V	Unclar pattern
**Tyrphostin A9**	X	V	V	V	Before and after fusion	V	V	V	All tested

**Table:** 

Compound	Suggested Mechanisms of Action
**Amiodarone**	Inhibition of fusion (lipid mixing)
**Amodiaquine**	Inhibition of fusion (lipid mixing)
**Bepridil**	Inhibition of fusion (lipid mixing)
**Calcimycin**	Inhibition of translation and unknown mechanisms
**Mitoxantrone**	Unknown mechanisms
**Niclosamide**	Inhibitor of viral internalisation and translation
**N-p-Tosyl-L-PCK**	Inhibitor of proteases important for different viruses
**Parthenolide**	Inhibition of translation and unknown mechanisms
**Raloxifene**	Inhibition of fusion (lipid mixing)
**Stattic**	Inhibition of lipid mixing, translation and unknown mechanisms
**Supercinnamaldehyde**	Unknown mechanisms
**Tyrphostin A9**	Inhibitor of viral internalisation and translation

## Dedication

We dedicate this paper to our friend, colleague, and collaborator, Marcus Dorner.

## Supplementary Materials

The following are available online at http://www.mdpi.com/1999-4915/11/2/176/s1, Figure S1: (A–C) Quantification of Western blots shown in Figure 2. Data are normalised to DMSO control cells and represent averages of three independent experiments. (D) Percentage of Renilla protein expression after treatment with indicated concentrations of compounds, normalised to expression in a DMSO treated control. Averages of two independent experiments are shown. E. Percentage of endogenous RNA expression after treatment with compounds, normalised to expression in a DMSO treated control (100%). Averages of two independent experiments are shown. Figure S2: Serum concentration of Niclosamide administered intraperitoneally (IP) (A) and Tyrphostin A9 administered orally (PO) (B) or IP (C) at the indicated time points after administration. Niclosamide was dosed at 10 mg/kg and Tyrphostin A9 at 1 mg/kg. Graphs are displayed both on a logarithmic (left panels) and on a linear (right panel) scale. Extrapolated parameters are displayed in the tables below each graph. Circled values represent tailpoint values. Figure S3: (A) Weight loss (relative to initial weight, 100%) over 7 days of treatment. (B) ZIKV RNA copies per ng of brain, liver, or lymph node tissue at 7 d.p.i. in the absence of treatment. (C) Percentage of CD3+CD8+ T cells infected by ZIKV 7 d.p.i. Table S1: List of compounds from the LOPAC and Prestwick libraries that inhibited more than 60% of SFV infection. Percentages of viability and percentages of inhibition compared to DMSO-treated controls are displayed. Compounds present in both libraries are highlighted in green. Table S2: The same compounds shown in Table S1, shown in order of potency against DENV-2. Percentages of viability and inhibition compared to DMSO-treated controls are shown. Compounds that blocked more than 60% of DENV-2 infection are highlighted in blue. Table S3: IC_50_, TC_50_, and Selectivity Index (SI) of the 25 compounds inhibiting both SFV and DENV-2 infection. The compounds with higher potency and selectivity index are shown in bold and were selected for further studies. Table S4: Statistical analysis of data shown in Figure 3B (Time of Addition experiment). Two-way Anova with Dunnett’s test for multiple comparisons.
