# Supplementary figures and images for: Identification of Broad-Spectrum Antiviral Compounds by Targeting Viral Entry
Source: Viruses. 2019 Feb 20;11(2):176. doi: 10.3390/v11020176 (PMC6410080; doi:10.3390/v11020176)

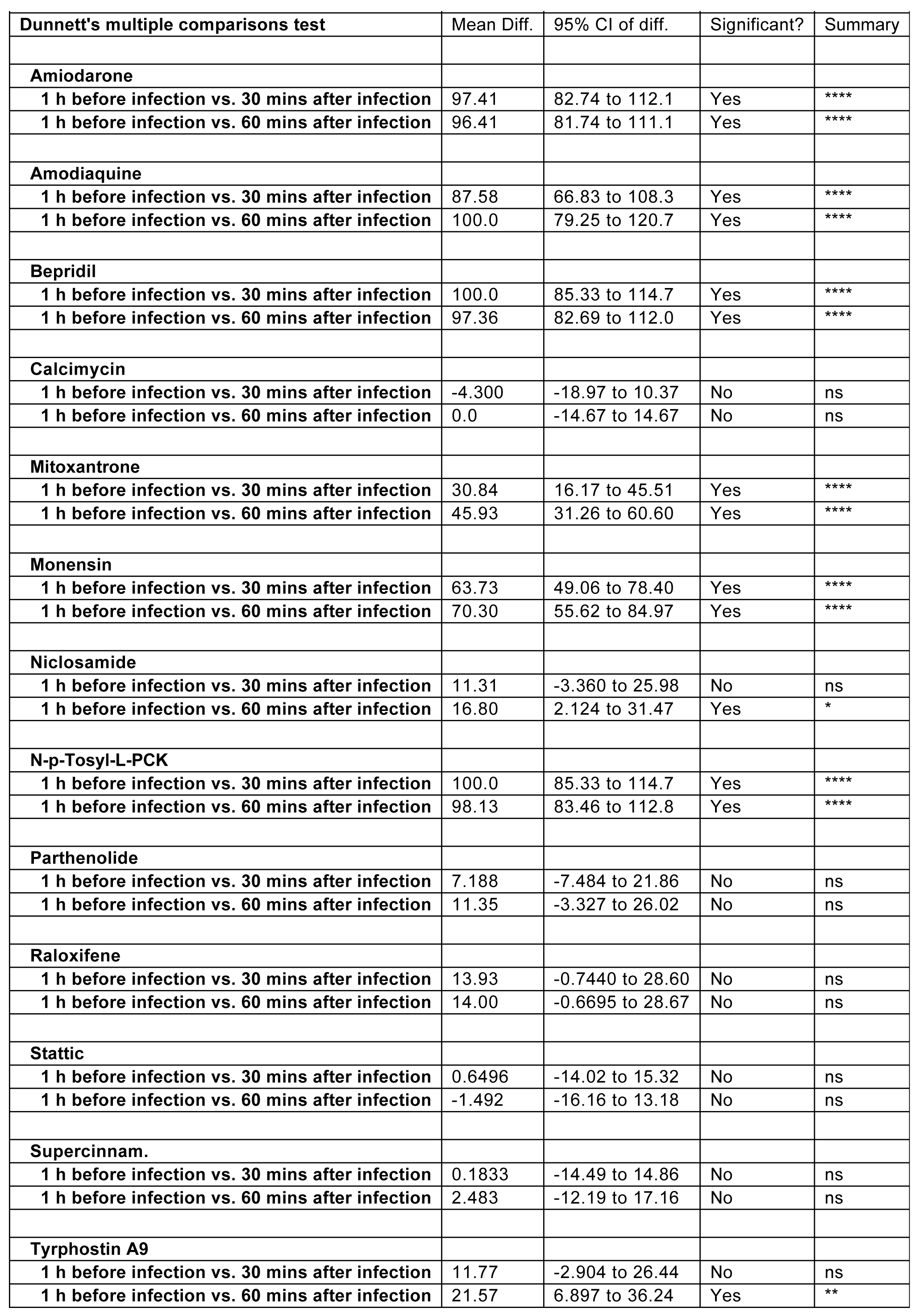

Supplement: Supplementary file 1 [file viruses-11-00176-s001.zip › Supplementary/Table S4.tif]

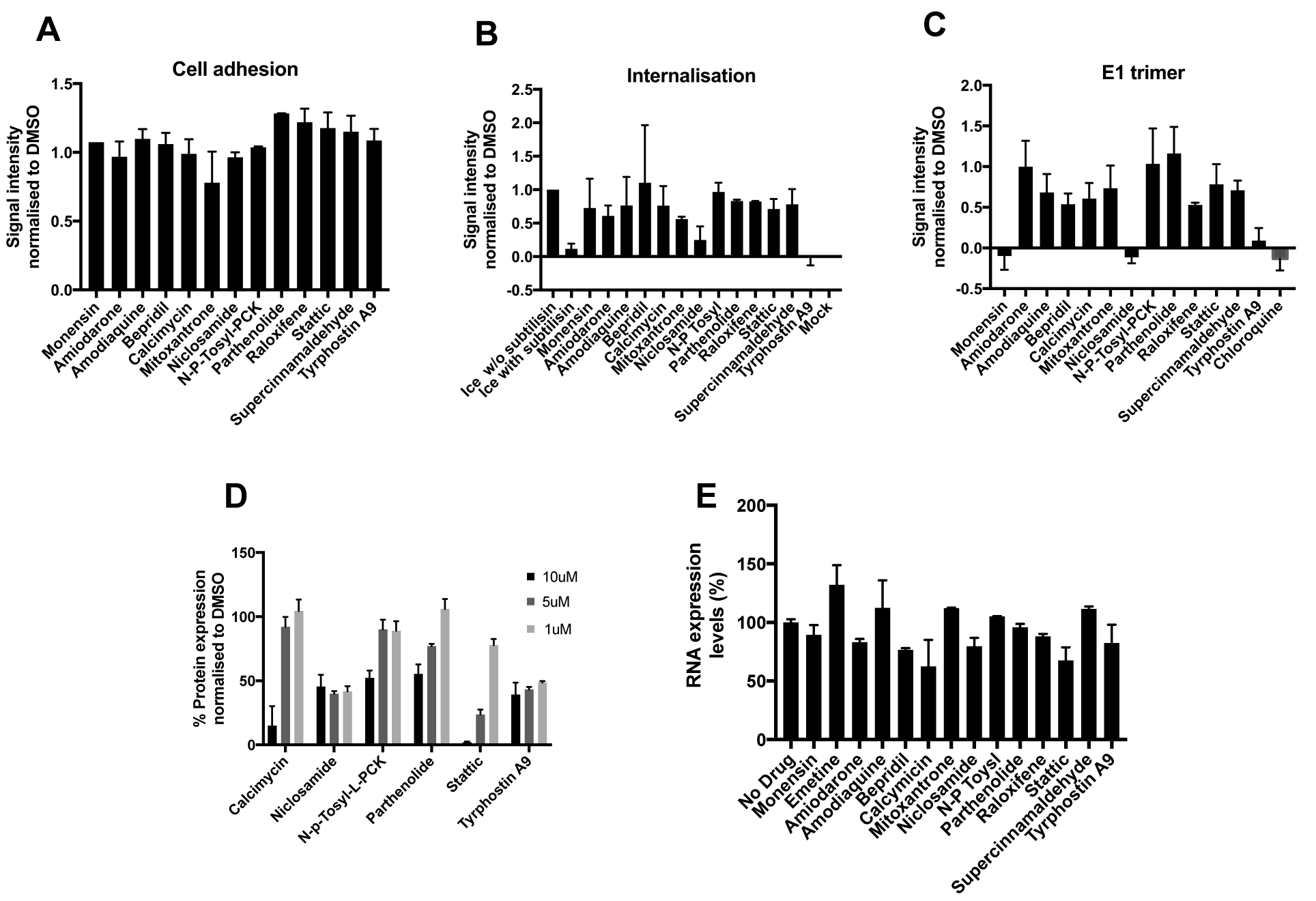

Supplement: Supplementary file 1 [file viruses-11-00176-s001.zip › Supplementary/SI 1.tif]

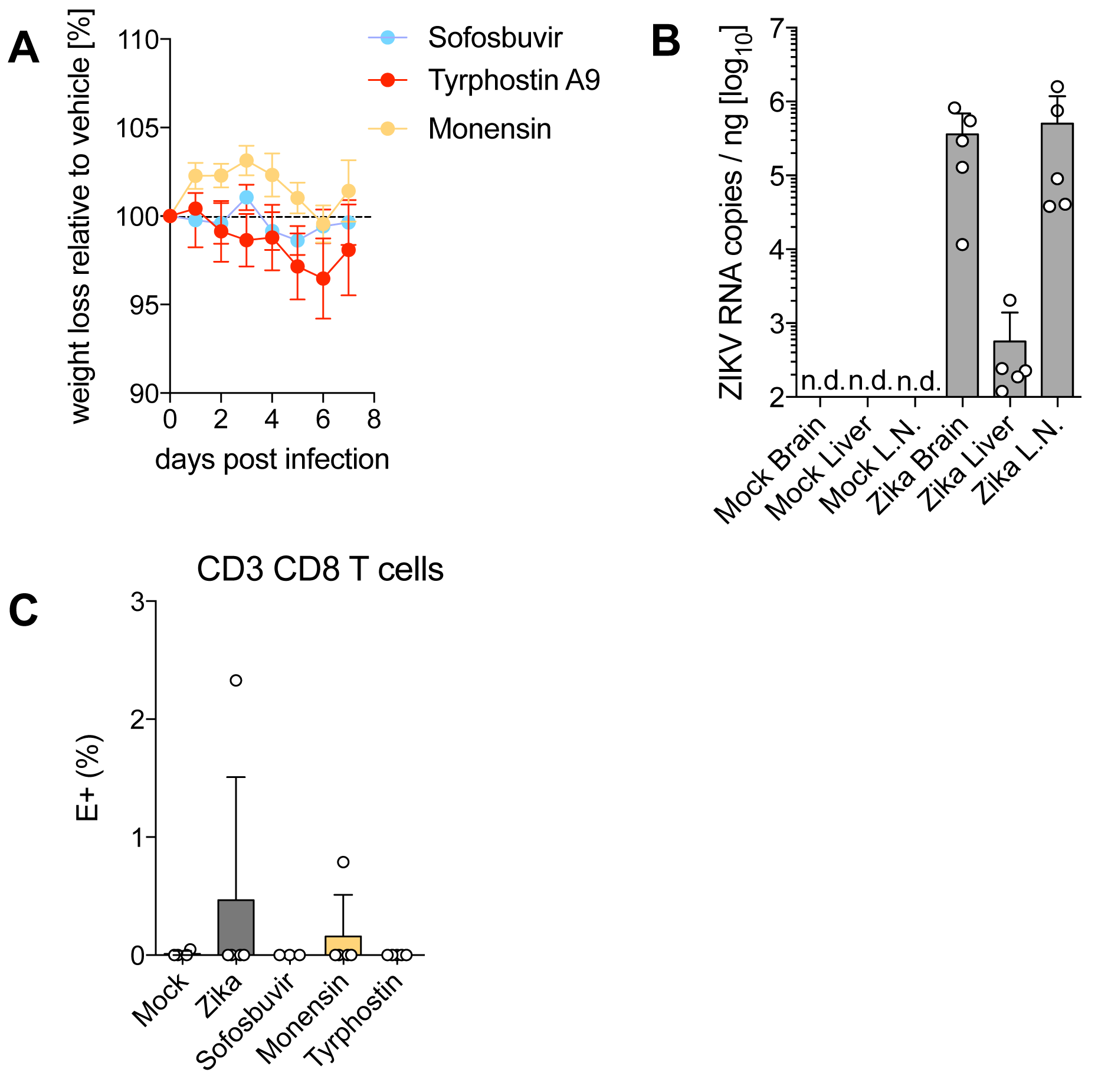

Supplement: Supplementary file 1 [file viruses-11-00176-s001.zip › Supplementary/SI 3.tif]

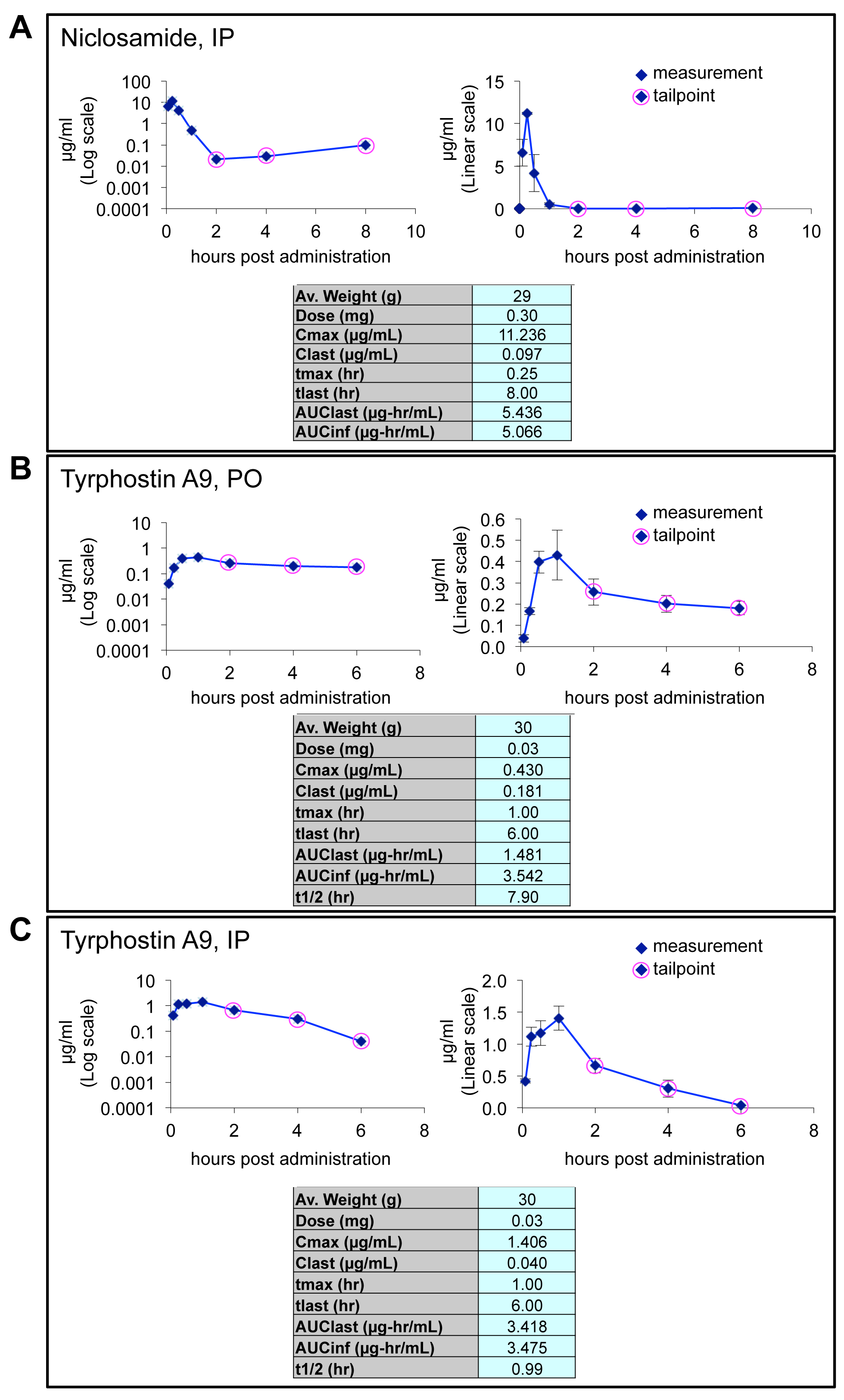

Supplement: Supplementary file 1 [file viruses-11-00176-s001.zip › Supplementary/SI 2.tif]
